# How quality of life is measured in studies of nutritional intervention: a systematic review

**DOI:** 10.1186/s12955-024-02229-y

**Published:** 2024-01-24

**Authors:** Raquel Clapés Pemau, Patricia González-Palacios, Kirk W. Kerr

**Affiliations:** 1 Abbott Nutrition, Granada, Spain; 2 Department of Nutrition and Food Science, University of Granada, Granada, Spain; 3 Biomedical Research Institute (IBS), Granada, Spain; 4 Abbott Nutrition, 2900 Easton Square Place, Columbus, OH 43219 USA

**Keywords:** Nutrition, Quality of life, Health outcomes

## Abstract

**Background:**

Nutrition care can positively affect multiple aspects of patient’s health; outcomes are commonly evaluated on the basis of their impact on a patient’s (i) illness-specific conditions and (ii) health-related quality of life (HRQoL). Our systematic review examined how HRQoL was measured in studies of nutritional interventions. To help future researchers select appropriate Quality of Life Questionnaires (QoLQ), we identified commonly-used instruments and their uses across populations in different regions, of different ages, and with different diseases.

**Methods:**

We searched EMCare, EMBASE, and Medline databases for studies that had HRQoL and nutrition intervention terms in the title, the abstract, or the MeSH term classifications “quality of life” and any of “nutrition therapy”, “diet therapy”, or “dietary supplements” and identified 1,113 studies for possible inclusion.We then reviewed titles, abstracts, and full texts to identify studies for final inclusion.

**Results:**

Our review of titles, abstracts, and full texts resulted in the inclusion of 116 relevant studies in our final analysis. Our review identified 14 general and 25 disease-specific QoLQ. The most-used general QoLQ were the Short-Form 36-Item Health Survey (SF-36) in 27 studies and EuroQol 5-Dimension, (EQ-5D) in 26 studies. The European Organization for Research and Treatment of Cancer Quality of life Questionnaire (EORTC-QLQ), a cancer-specific QoLQ, was the most frequently used disease-specific QoLQ (28 studies). Disease-specific QoLQ were also identified for nutrition-related diseases such as diabetes, obesity, and dysphagia. Sixteen studies used multiple QoLQ, of which eight studies included both general and disease-specific measures of HRQoL. The most studied diseases were cancer (36 studies) and malnutrition (24 studies). There were few studies focused on specific age-group populations, with only 38 studies (33%) focused on adults 65 years and older and only 4 studies focused on pediatric patients. Regional variation in QoLQ use was observed, with EQ-5D used more frequently in Europe and SF-36 more commonly used in North America.

**Conclusions:**

Use of QoLQ to measure HRQoL is well established in the literature; both general and disease-specific instruments are now available for use. We advise further studies to examine potential benefits of using both general and disease-specific QoLQ to better understand the impact of nutritional interventions on HRQoL.

**Supplementary Information:**

The online version contains supplementary material available at 10.1186/s12955-024-02229-y.

## Introduction

Nutrition interventions play a crucial role in the management of a wide range of physiological and pathological conditions. The link between proper nutrition and good health status has been transformed from a scientific research field to a focal point in institutions and governments. For example, the Rockefeller Foundation and the American Heart Association have created the Food is Medicine Research Initiative, with a goal to support and promote nutrition as a preventive tool and as part of the treatment for various conditions, such as diabetes, cardiovascular diseases, cancer, renal diseases, arthritis, mental health, and neurological disorders [1]. Additionally, evidence from numerous studies supports the effectiveness of nutrition therapy in the management of diabetes [2, 3]. A healthy diet and moderate physical activity can reduce the risk of developing diabetes by 58% [4]. Research has also shown that nutritional supplements and dietary interventions can reduce the risks of negative cardiovascular outcomes [5, 6].

Nutrition interventions are crucial for improving the nutritional status of an individual who is malnourished or at risk of malnutrition. Malnutrition has been linked to reduced immune function, increased infection rates, prolonged hospitalization, high medical expenditure, and increased mortality rates [7]. Disease-related malnutrition is prevalent in conditions such as cancer, with a prevalence ranging from 50–80% due to disease-related anorexia and various symptoms associated with both the disease and its treatment [8]. Studies across multiple populations have shown the positive impact of improving nutritional status on an individual’s overall health [9, 10]. Because of their potential to impact multiple aspects of patient health, nutrition interventions should not only be evaluated based on their impact on specific illnesses, but also on their effect on the individual's health-related quality of life (HRQoL).

Quality of life (QoL) is defined by the World Health Organization (WHO) as an individual's perception of their position in life in the context of the culture and value systems in which they live and in relation to their goals, expectations, standards, and concerns [11]. It is affected in complex ways by the person's physical health, psychological state, level of independence, social relationships, and how the person relates to key features of their environment. Given the complexity of the concept, the assessment of QoL is challenging and requires multiple measures to capture subjectivity and multidimensionality [12]. Various instruments have been developed to measure the above domains, but none are recognized as the "gold standard" [13]. Quality of Life Questionnaires (QoLQ) are extensively employed as HRQoL instruments in clinical or experimental contexts and they can be used to determine the patient-self-perceived health state and/or as part of a cost-effectiveness analyses for health economic evaluation [14].

QoLQ can be either general for several conditions or disease-specific - designed and validated for the assessment of QoL in specific populations. General QoLQ facilitate QoL measurement across diseases and interventions and enable policy evaluation. Disease-specific QoLQ instruments allow researchers to evaluate changes in health-related QoL aspects of a particular illness. The choice of instrument is often based on factors such as the purpose of the study, the studied population, available resources, and subsequent data handling [13].

Nutrition has the potential to enhance individual’s QoL, and therefore, should be assessed in nutrition interventions. However, the selection of QoLQ can be complex, considering the variety of study characteristics and the lack of guidelines or consensus on the most suitable tools to be used in nutrition interventions. This systematic review summarizes the available evidence about the use of QoLQ in the context of nutrition interventions based on its characteristics across different populations, diseases, and regions with the objective to shed light on the choice of QoLQ in future nutrition research.

## Methods

### Search strategy

A systematic review of the literature was conducted of studies evaluating the impact of nutritional interventions on QoL. Articles were identified by searching the EMCare, EMBASE, and Medline databases. The search strategy identified articles that included “Quality of Life” and “nutrition intervention” terms in the title or abstract or were classified by the MeSH terms “quality of life" and any of “nutrition therapy”, “diet therapy”, or “dietary supplements”. See Supplemental Fig. 1 (see Additional file 1) for complete Boolean logic used to search all databases. Articles in English published up to September 2022 in journal articles reporting on randomized controlled trials, multicenter studies, clinical trials, comparative studies, observational studies, and case reports were included. In addition to the electronic search, a manual search of review articles was conducted, resulting in the addition of seven articles. An additional four trials were identified through cost-effectiveness studies based on original trials captured in our search.

**Fig. 1:**
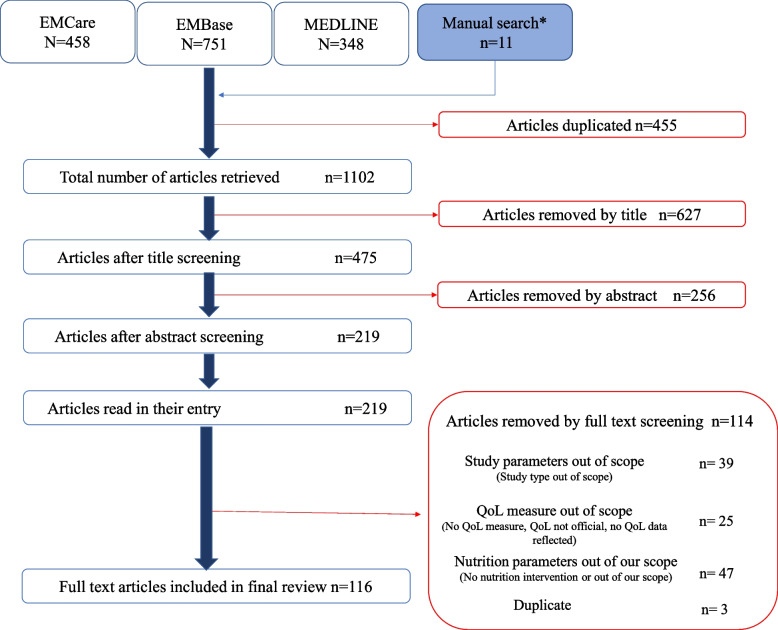
PRISMA diagram outlining screening of articles for inclusion in this review

### Inclusion and exclusion criteria

Articles were considered eligible for inclusion if they used a validated measure of patient QoL (patient reported or other) or were part of an initiative to develop or validate a QoL measure. Additionally, the primary study intervention needed to be a nutrition intervention, where nutrition intervention was broadly defined to include oral nutritional supplements, nutrition education or counseling, enteral nutrition, parenteral nutrition, or other activities to improve the nutrition consumed by study patients [15]. Studies in which the nutritional intervention was part of a broader quality improvement program or protocol shift that included a change in nutrition care, such as an enhanced recovery after surgery protocol with a nutrition component, were included. Studies were excluded if the primary study intervention was not nutrition or nutrition-related, or if the study used a QoL measure that is not accepted by the scientific community or QoLQ instruments that were not validated. All studies identified by the initial search were reviewed by the authors to determine if the study fully met inclusion criteria. Study titles and abstracts were assessed by all authors to identify relevant studies, with studies not meeting inclusion criteria eliminated. Full text of the remaining studies was reviewed to determine if inclusion criteria were met. Disagreements on inclusion or exclusion were resolved by group discussion and consensus.

### Data collection

Data was collected by the group on each study, reviewing the full text to identify nutrition intervention, QoL instrument used, patient population, and medical condition or pathology addressed by the intervention. Information from each study was input to a custom Excel spreadsheet developed by the authors. Questionnaires were classified by whether they measured QoL in a general or specific population. A questionnaire was classified as measuring QoL in a specific population if its intended use was limited to a group identified by age, sex, or medical condition, whereas general questionnaires could be used regardless of patient age, sex, or medical condition. For population-specific questionnaires, the target population was recorded. We also collected data on the locations where nutrition intervention studies using QoLQ were conducted.

Collected data are summarized in Supplemental Materials Table 1 (see Additional file 1).

## Results

Results from the authors’ review are summarized in the PRISMA diagram (Fig. 1). Our initial search yielded 1,102 studies. Review of titles, abstracts and full-text resulted in 116 studies being included in our analysis. A total of 39 QoLQ were identified in the 116 included studies. 72 studies used a general (62%) and 52 used a disease-specific or population-specific QoLQ (45%). We identified 14 general QoLQ, and 25 disease- or population-specific QoLQ; 10 of the disease-specific QoLQ focused on various types of cancer. Summary of all questionnaires identified, classified, and briefly described can be found in Table 1.

**Table 1:** Summary of reviewed questionnaires

Questionnaire	Target population	Definition	Frequency of use	References
SF-36	General	36-item self-report measure of HRQoL with physical health and mental health component scores derived from eight subscales measuring different domains of health-related quality of life: physical functioning, role-physical, bodily pain, general health, vitality, social functioning, role-emotional, and mental health. [16]	27	[17–43]
SF-12	General	Shorter version of the SF-36. [44]	3	[45–47]
EQ-5D-3L	General	Self-reported measure of HRQoL using 3 levels to describe health across five dimensions (Mobility, Usual Activities, Self-care, Pain & Discomfort, and Anxiety & Depression). [48]	15	[35, 49–62]
EQ-5D-5L	General	Self-reported measure of HRQoL using 5 levels to describe health across five dimensions (Mobility, Usual Activities, Self-care, Pain & Discomfort, and Anxiety & Depression) [48]	11	[63–73]
EQ-5D VAS	General	Self-rated health scale included in the EQ-5D questionnaire (both 3L and 5L version) that allows individuals to rate their current health status on a scale from 0 to 100. The VAS score is considered a global measure of health status [48]	1	[74]
WHOQoL-BREF	General	26-items HRQoL scale consisting of four domains (physical health, psychological, social, and environmental). Each domain is scored on a 5-point scale from 0 to 100, with higher scores indicating better QoL [75]	6	[76–81]
PedsQoL	General	Instrument for assessing patients and parents’ perceptions of HRQoL in pediatric patients with chronic health conditions. Consists of a 15-item core measure of global HRQoL AND eight supplemental modules assessing specific symptoms or treatment domains [82]	2	[83, 84]
AQoL-8D	General	QoL questionnaire based in AQoL-6D questionnaire which adds explicit dimensions for self-worth and happiness and expands the items in mental health [85–87]	2	[88, 89]
AQoL-6D	General	QoL questionnaire based in AQoL-4D questionnaire were. pain and coping dimensions are added and mental health and independent living items are increased from 3 to 4 items [86, 90]	1	[91]
AQoL-4D	General	QoL questionnaire developed by Australian researchers, is a multi-attribute utility HRQoL instrument. While it can be used to measure HRQoL alone, its main purpose is to measure the ‘utility’ of health states. This version contains 4 dimensions (independent living, social relationships, physical senses and psychological wellbeing [86, 92])	1	[93]
NHP	General	Self-administered questionnaire that measures an individual's physical, emotional, and social well-being in the context of their current health status [94]	1	[95]
POMS2	General	Questionnaire used to evaluate transient feelings and mood in individuals aged 13 years and above. It has evolved from the original POMS and is applicable in multiple settings, such as monitoring mood changes during interventions and assessing the impact of physical disease on psychological functioning [96]	1	[97]
PROMIS SF	General	Standardized set of measures developed by the National Institutes of Health (NIH) to assess various aspects of health-related quality of life (HRQoL) from the patient's perspective [98]	1	[99]
SIP	General	Behaviorally based measure of health status used to assess a person's perception of their health status with respect to their disease impact [100]	1	[101]
CANCER QOLQ				
EORTC-QLQ-C30	Cancer	HRQoL questionnaire for individuals with cancer incorporating five functional scales (physical, role, cognitive, emotional, and social), three symptom scales (fatigue, pain, and nausea and vomiting), and a global health and quality-of-life scale [102]	27	[19, 68, 103–127]
EORTC-QLQ-OES18	Cancer (esophageal symptoms)	EORTC questionnaire module used with the EORTC-QLQ-C30 to assess QoL in patients with esophageal cancer undergoing any single or combination of treatments including: esophagectomy, chemoradiation, endoscopic palliation or palliative chemotherapy and/or radiotherapy [128]	4	[65, 109, 117, 126]
EORTC-QLQ-H&N35	Cancer (Head and neck)	EORTC questionnaire module used with the EORTC-QLQ-C30 to assess QoL in patients with head and neck cancer undergoing any single or combination of treatments (i.e. surgery, radiotherapy and chemotherapy) [129]	2	[110, 126]
EORTC-QLQ-BR23	Cancer (Breast)	EORTC questionnaire module used with the EORTC-QLQ-C30 to assess QoL in patients with breast cancer undergoing any single or combination of treatments (i.e. surgery, chemotherapy, radiotherapy and endocrine treatment) [129]	1	[119]
EORTC-QLQ-PAN26	Pancreatic cancer	EORTC questionnaire module used with the EORTC-QLQ-C30 to assess QoL in patients with pancreatic cancer. Includes 26 items related to disease symptoms, treatment side-effects and emotional issues specific to pancreatic cancer [129]	1	[124]
FACT-G	Cancer	Questionnaire designed to measure four domains of HRQOL in cancer patients: Physical, social, emotional, and functional well-being [130]	2	[46, 131]
FACT-G7	Cancer	The FACT-G7 (Functional Assessment of Cancer Therapy-General 7 item) is a shortened version of the FACT-G questionnaire [132]	1	[133]
FAACT	Anorexia and Cachexia by Cancer	QoL questionnaire for both cancer and HIV-infected patients with anorexia/cachexia [134]	1	[135]
FACT-B	Cancer (Breast)	Self-report instrument designed to measure QoL in patients with breast cancer. The FACT-B consists of the FACT-General (FACT-G) plus specific concerns related to breast cancer) [136]	1	[131]
FACT-C	Cancer (colorectal)	Self-report instrument designed to measure QoL in patients with colorectal cancer. The FACT-C consists of the FACT-General (FACT-G) plus specific concerns related to colorectal cancer [137]	1	[46]
OTHER DISEASE-SPECIFIC QOLQ				
SGRQ	Obstructive airways disease	Self-administered HRQoL to measure three components: Symptoms, Activity, and Impacts. Scores ranging from 0 to 100 with higher scores indicating less QoL. It is valid for COPD, bronchiectasis, and asthmatic populations. [138]	3	[139–141]
MLHFQ	Heart failure	Self-administered questionnaire that measures the impact of heart failure on QoL using a 21 item six-point Likert scale. It provides a total score ranging from 0 to 105 with higher scores indicating worse quality of life. [142]	2	[143, 144]
PAC-QoL	Constipation	Self-administered questionnaire used to assess the impact of constipation on a patient's QoL. It consists of 28 items across four domains(physical discomfort, psychosocial discomfort, worries and concerns, and satisfaction.) [145]	2	[146, 147]
QUALIDEM	Dementia	Two-versions QoLQ to be used in different stages of dementia: 37-item version for people with mild to severe dementia covering nine domains of QoL (care relationship, positive affect, negative affect, restless tense behavior, positive self-image, social relations, social isolation, feeling at home, and having something to do) and the 18- items version for people with very severe dementia (deleting domains of positive self-image, feeling at home, and having something to do). [148]	2	[149, 150]
CLDQ	Chronic liver disease	29- items QoL questionnaire for assessing the HRQoL in individuals with chronic liver disease covering 8 domains (abdominal symptoms, fatigue, systemic symptoms, activity, emotional function, worry). [151]	1	[152]
IWQOL-Lite	Weight	Shortened version of the Impact of Weight on QoL (IWQOL) questionnaire. It is a 31-item instrument consisting of five scales: Physical Function, Self-Esteem, Sexual Life,, Public Distress, and Work. [153]	1	[17]
KDQOL-SF	Chronic kidney disease	Shorter version, self-report measure developed for individuals with kidney disease and those on dialysis. It includes kidney disease-targeted items, (effects of the disease of activities of daily living, work status, and social interaction) physical and mental health items) and 1 overall health rating item. [154]	1	[70]
KOOS QoL	Knee Injury and Osteoarthritis	Extension of the WOMAC Osteoarthritis Index to evaluate subjects with knee injury and osteoarthritis. It contains 5 scored subscales: Pain, other Symptoms, Function in daily living (ADL), Function in Sport and Recreation (Sport/Rec), and knee-related (QOL). [155]	1	[156]
MiniAQLQ	Asthma	MiniAQLQ is a shortened version of the AQLQ(S) questionnaire. It consists in 15 questions covering symptoms, activities, emotions, and environment domains. [157]	1	[158]
MSQoL-54	Multiple sclerosis	HRQoL measure that combines both generic (based in SF-36 questionnaire) and MS-specific items (such as fatigue, cognitive function, etc.) into a single instrument divided in physical function, role limitations-physical, role limitations-emotional, pain, emotional well-being, energy, health perceptions, social function, cognitive function, health distress, overall quality of life, and sexual function subscales. [159, 160]	1	[161]
PDQ-39	Parkinson	Tool that assesses how often people with Parkinson's experience difficulties across 8 dimensions of daily living including relationships, social situations, and communication. [162]	1	[163]
QoL-B	Non-cystic fibrosis (CF) bronchiectasis	Self-administered measure assessing symptoms, functioning and HRQoL of patients with non-cystic fibrosis and bronchiectasis. It contains 37 items on 8 scales (Respiratory Symptoms, Physical, Role, Emotional and Social Functioning, Vitality, Health Perceptions and Treatment Burden). [164]	1	[165]
QUALEFFO-41	Vertebral deformities	Questionnaire for measuring QoL in patients with vertebral osteoporosis. It consists of questions and visual analogue scales in pain, activities of daily living, jobs around the house, mobility, leisure and social activities, general health perception and mood domains. [166]	1	[52]
RV-DQOL	Diabetes	Questionnaire with three major domains: diabetes life satisfaction scale (QoL satisfy), disease impact scale (QoL impact), disease related worries scale (QoL worry) and a general health questionnaire (GHQ). This instrument has been used in numerous studies in both type I DM and type 2 DM patients. [167]	1	[168]
SWAL-QOL	Dysphagia	SWAL-QOL, a disease-specific measure of quality of life and quality of care for patients with oropharyngeal dysphagia. It assesses 10 QoL concepts; eight of which are dysphagia-related QoL (food selection, burden, mental health, social functioning, fear, eating duration, eating desire, communication) and two pertaining to general QoL (sleep and fatigue). [169]	1	[78]

*Abbreviations*: *AQoL-4D* Assessment of Quality of Life-4 Dimension, *AQoL-6D* Assessment of Quality of Life-6 Dimension, *AQoL-8D* Assessment of Quality of Life-8 Dimension, *CLDQ* Chronic Liver Disease Questionnaire, *EORTC* European Organization for Research and Treatment of Cancer, *EORTC-QLQ-BR23* EORTC-Breast Cancer Module, EORTC-QLQ-C30. EORTC- General Cancer Questionnaire, *EORTC-QLQ-H&N35* EORTC- Head & Neck Cancer Module, *EORTC-QLQ-OES18* EORTC- Oseophageal Cancer Module, *EQ-5D-VAS* EuroQol– 5 Dimension Visual Analog Scale, *EQ-5D-3L* 3-level EuroQol– 5 Dimension, *EQ-5D-5L* 5-level EuroQol– 5 Dimension, *FAACT* Functional Assessment of Anorexia/Cachexia Treatment, *FACT-B* The Functional Assessment of Cancer Therapy – Breast, *FACT-C* Functional Assessment of Cancer Therapy – Colorectal, *FACT-G* Functional Assessment of Cancer Therapy – General, *FACT-G7* Functional Assessment of Cancer Therapy – General – 7 Item Version, *IWQOL-Lite* Impact of Weight on Quality of Life-Lite, *KDQOL-SF* Kidney Disease Quality of Life Short Form, *KOOS* Knee Injury and Osteoarthritis Outcome Score, *MiniAQLQ* Mini Asthma Quality of Life Questionnaire, *MLHFQ* Minnesota Living With Heart Failure Questionnaire, *MSQoL-54* Multiple Sclerosis Quality of Life-54, *NHP* Nottingham Health Profile, *PAC-QoL* Patient Assessment Of Constipation Quality of Life, *PAN26* EORTC Pancreatic Cancer Module, *PDQ-39* The Parkinson's Disease Questionnaire, *PedsQoL* Pediatric Quality of Life Inventory, *POMS2* Profile of Mood States Second Edition, *PROMIS SF* Patient Reported Outcomes Measurement Information System – Short Form, *QoL-B* Quality of Life Questionnaire-Bronchiectasis, *QUALEFFO-41* Quality of Life Questionnaire of the European Foundation for Osteoporosis, *QUALIDEM* Quality of Life for People with Dementia, *RV-DQOL* Revised Version of Diabetes Quality of Life Instrument, *SF-12* Short-Form 12-Item Health Survey, *SF-36* Short-Form 36-Item Health Survey, *SGRQ* St. George's Respiratory Questionnaire, *SIP* Sickness Impact Profile, *SWAL-QOL* Swallowing Quality of Life questionnaire, *WHOQoL-BREF* World Health Organization Quality of Life Scale-BREF

**Fig. 2:**
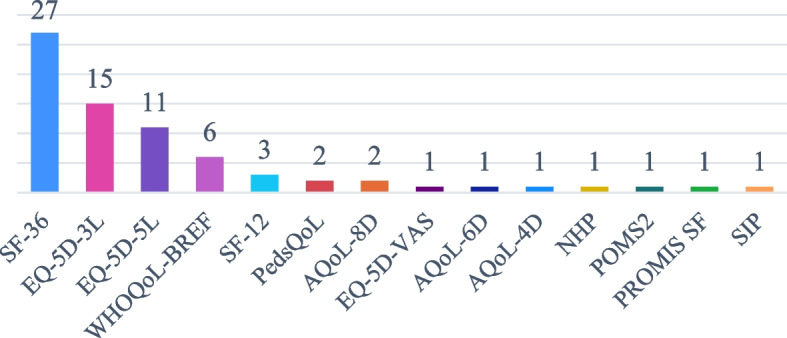
General QoLQ usage (absolute frequency) in nutrition interventions

 Of the 14 general QoLQs identified in our search, a large difference in the frequency of use among them was detected, as illustrated in Fig. 2. The Short Form series questionnaires (SF series including SF-36 and SF-12) were the most frequently used questionnaires, appearing in 30 studies. The EQ-5D questionnaires, including EQ-5D-3L and EQ- 5D-5L (also one study used part of the questionnaire, the EQ-5D-VAS), were used in 26 studies and were the second most frequently used general questionnaire.

**Fig. 3:**
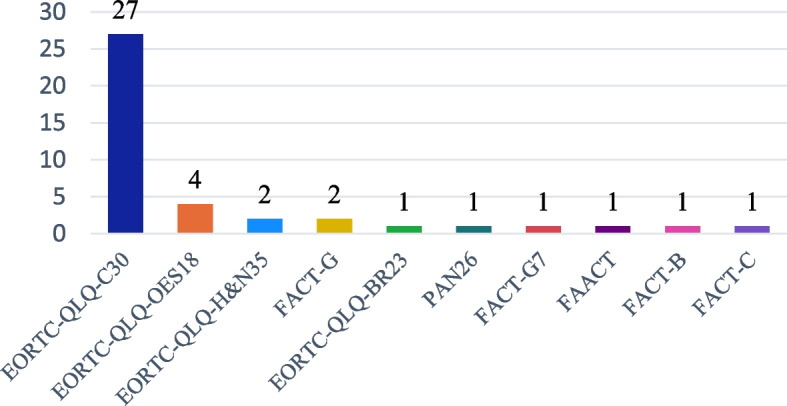
Cancer QoLQ usage (absolute frequency) in nutrition interventions

When examining the 25 disease-specific QoLQ, we found that a wide range of tools designed for specific pathologies, but cancer was the only disease with multiple disease-specific QoLQ and was the focus of our analysis of these instruments. To better understand the different tools used to assess QoL in cancer, Fig. 3 shows all the cancer-related QoLQ and their frequency of use in the nutrition intervention studies analyzed. The EORTC family of questionnaires (including EORTC-QLQ-C30, EORTC-QLQ-OES18, EORTC-QLQ-BR23, EORTC-QLQ-H&N35 and EORTC-QLQ-PAN26) were the most frequently used cancer-specific QoLQ, appearing in 28 studies.

Although most studies used only one QoLQ, 16 studies used multiple QoLQ. Six studies combined a general QoLQ with a disease-specific QoLQ. Eight studies used two disease-specific (cancer) QoLQ. One study used two general QoLQs, the SF-36 and EQ-5D, to assess QoL. Finally, we identified a study that used 3 questionnaires, 2 disease-specific, and 1 general QoLQ, to assess QoL during the intervention.

**Fig. 4:**
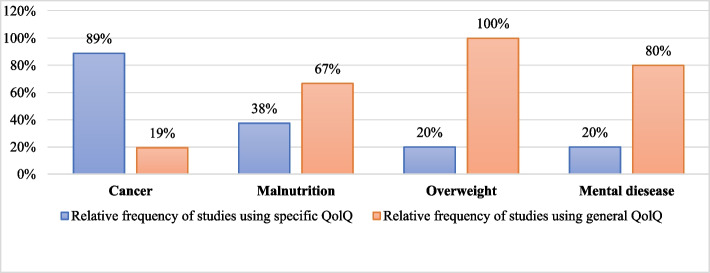
Relative frequency of studies using general and/or disease-specific QoLQ among the most prevalent pathologies observed

Many pathologies were being treated in the nutrition interventions using QoLQ included. Cancer was the most frequently studied condition, followed by malnutrition. To determine whether there was a trend in the use of disease-specific and/or general questionnaires based on the pathology, we categorized the questionnaires used in the most prevalent pathologies. The relative frequency (%) of each group of QoLQ usage based on the pathology can be seen in Fig. 4. As observed, only in studies related to cancer was the use of disease-specific QoLQ is more widespread than general QoLQ, despite availability of disease-specific questionnaires for all the analyzed pathologies.

Next, we analyzed which questionnaires were used for each pathology: fifteen different QoLQ were used in the 36 cancer-related studies included in our review. Thirty-two (88%) of these studies included at least one cancer-specific QoLQ. Conversely, malnutrition studies mostly used general QoLQ, such as EQ-5D and SF-36. Studies focusing on overweight used SF-36 more often than a disease-specific questionnaire (e.g., IWQOL-Lite). Table 2 shows the most frequently studied pathologies within the papers included in our review, the QoLQ used in each, and the number of studies where the QoLQ mentioned was used.

**Table 2:** Pathology in nutrition intervention studies and questionnaires to evaluate quality of life

Pathology (nº studies)	Questionnaire						Studies using QoLQ
**Cancer (36)**	EORT-QLQ-C30						27
	EORTC-QLQ-OES18						4
	SF-36						3
	EORTC-QLQ-H&N35	FACT-G					2
	EQ-5D-5L						
	FACT-G7	EQ-5D-3L	NHP	PAN26	EORTC-QLQ-BR23		1
	FACT-B	SF-12	FACT-C	FAACT			
**Malnutrition (24)**	EQ-5D-3L						5
	EQ-5D-5L						
	EORTC-QLQQ-C30						4
	SF-36						3
	MLHFQ						2
	NHP	SGRQ	KDQOL-SF				1
	AQoL-6D	SF-12					
**Overweight (5)**	SF-36						3
	PedsQL						1
	WHOQoL-BREF						
	IWQOL-Lite						
**Mental diseases (5)**	AQoL-8D						2
	EORTC-QLQ-C30	WHOQoL-BREF					1
	QLQ-BR23	POMS2					

Some studies use more than 1 QoLQ, therefore, number of questionnaires may not sum number of studies in each pathology

We also examined the use of QoLQ in specific populations. Most of the studies were carried out in adult populations, with only four studies (3% of the total) focused on pediatric populations. The PedsQL questionnaire, exclusively designed for the pediatric population appeared only in 2 studies despite being an established instrument for measuring QoL in pediatric research. Research in older adults was also limited, finding only 38 studies (33% out of total) carried out in population older than 65 years. Malnutrition was the pathology most frequently studied in this population. Table 3 summarizes the pathologies and QoLQ in these age-specific populations.

**Table 3:** Population-specific quality of life studies in nutrition intervention studies

Age group	Nº of studies	Pathology (Nº of studies related with)	QoLQ used (Nº of studies where used)
Pediatric	4	Mental issue (1)	WHOQoL-BREF
		Overweight (2)	PedsQL (1), SF-36 (1)
Elderly	38	Nonspecific (1)	PedsQL
		Malnutrition (13)	QUALIDEM (1), EQ-5D-5L (4), EQ-5D-3L (5), SF-36 (2), AQoL-6D(1)
		Alzheimer (1)	WHOQoL-BREF
		Bone fracture (1)	EQ-5D-3L
		Cancer and malnourished (1)	EORTC-QLQ-C30
		Chronic kidney disease (1)	WHOQoL-BREF
		Diabetes (1)	EQ-5D-3L
		Heart failure (1)	SF-36
		Mental disease (1)	POMS2
		Overweight (1)	SF-36, IWQOL-Lite
		Pain And Rheumatic disease (1)	KOOS QoL
		Pulmonary disease (1)	SGRQ
		Sarcopenia (1)	SF-36
		None specific (14)	EQ-5D-5L (3), EQ-5D-3L (4), SF-36 (5), AQoL-4D(1), QUALIDEM (1)

**Fig. 5:**
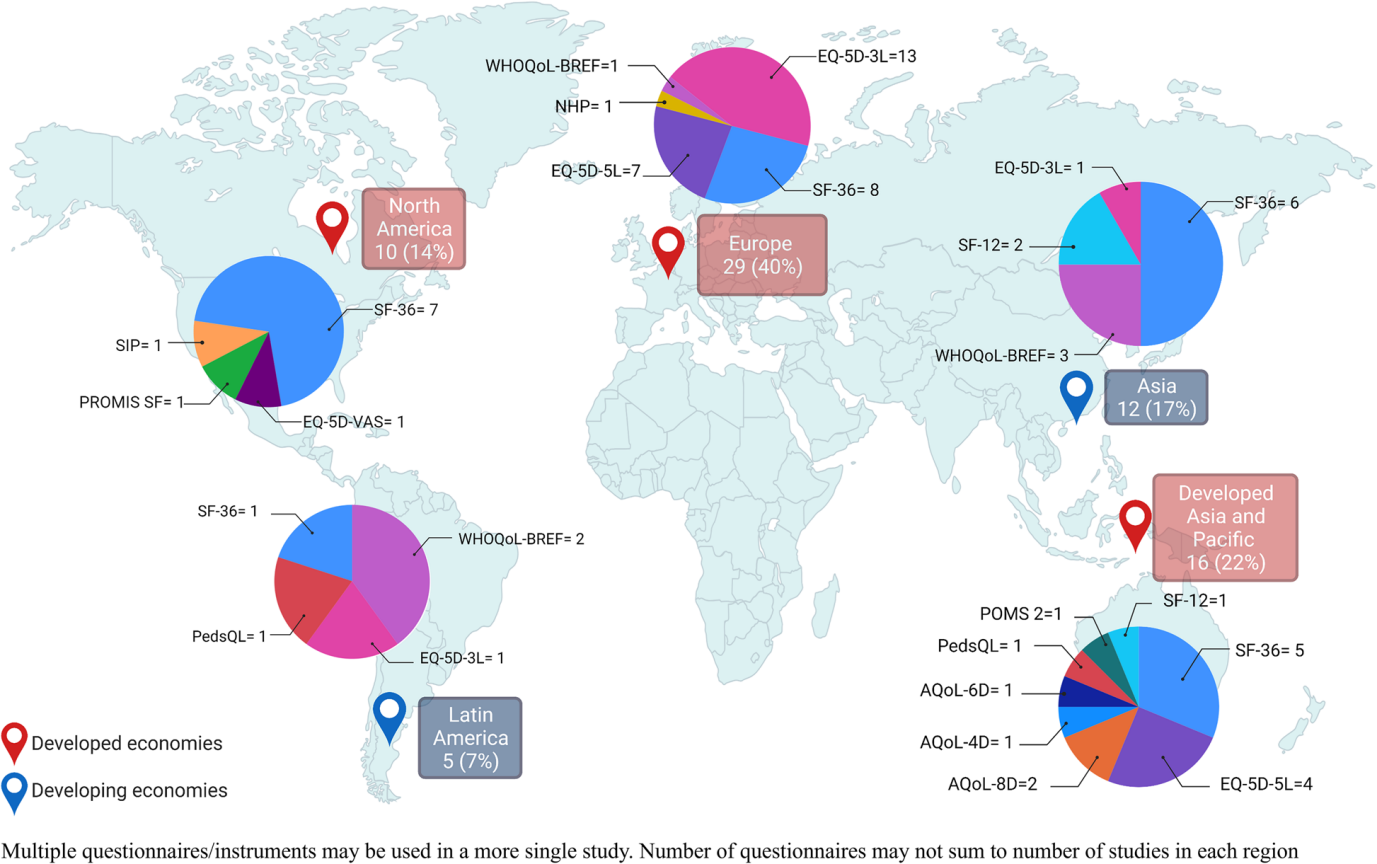
Distribution of studies using general QoLQ by regions defined by the United Nations in the The World Economic Situation and Prospects 2020 (UN2020) (labels) and classification of type of general QoLQ used in each region (circle graphs)

Finally, we evaluated the regions in which QoLQ were used. Regions were categorized as developed or developing economies following the UN2020 Classification of Developed Countries [170]. Twenty-nine studies using a general QoL were conducted in Europe (40% of the total studies using general QoLQ), followed by sixteen studies conducted in Developed Asia and Pacific (22%), twelve in Asia (17%), ten in North America (14%), and five in Latin America (7%). In Europe, the EQ-5D questionnaires were the most frequently used (20 studies) followed by the SF-36 (8 studies). In the Developed Asia Pacific region, SF questionnaires were the most used (6 studies), closely followed by the three versions of the AQoL questionnaire (4 studies) and the EQ-5D (4 studies). In Asia and North America, the SF questionnaires were most frequently used (8 and 7 respectively). Figure 5 summarizes the use of general QoLQ and number of studies that appeared in each region.

**Fig. 6:**
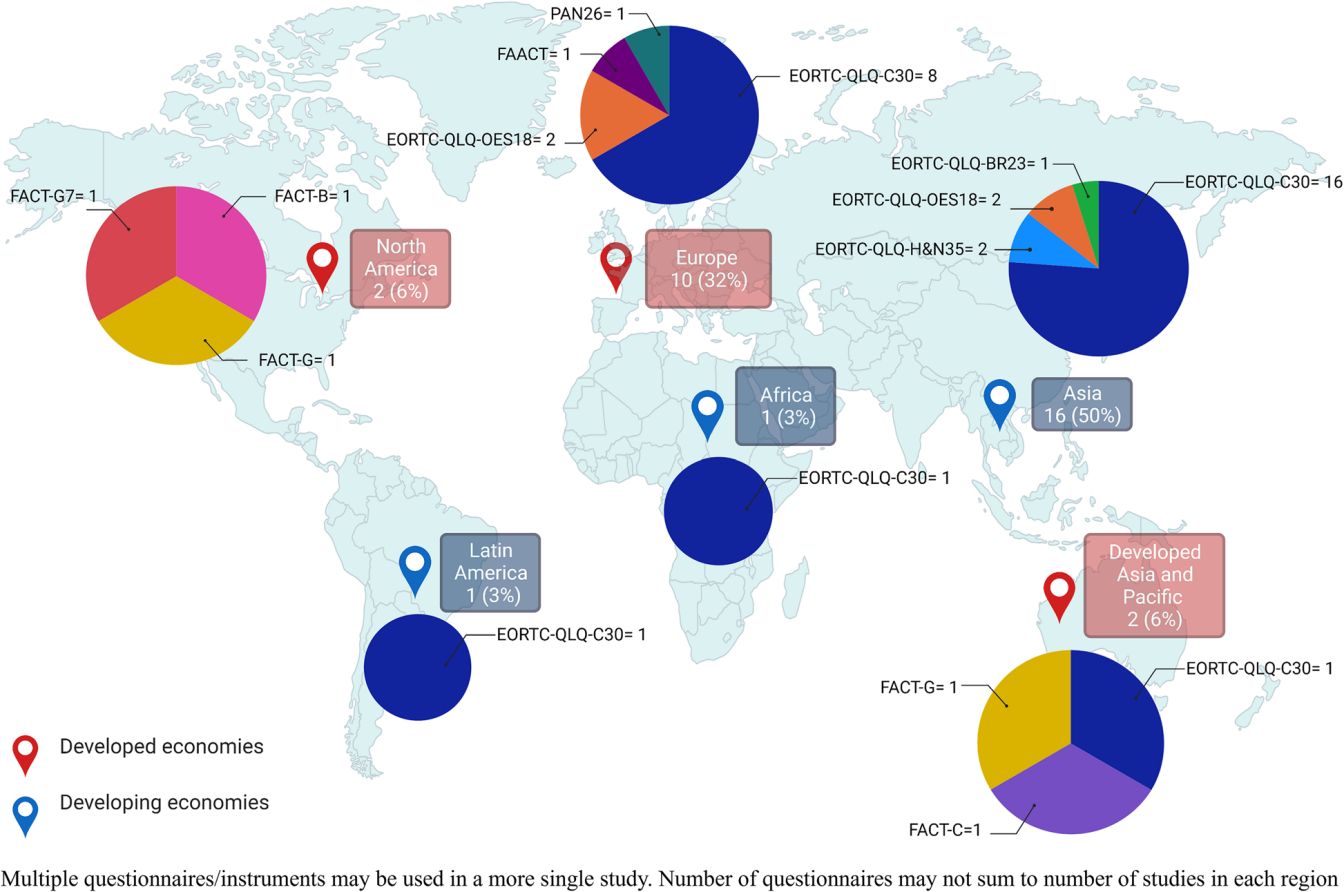
Distribution of studies using cancer QoLQ by regions defined by the UN2020 classification (labels) and classification of type of cancer QoLQ used in each region (circle graphs)

Half of studies using cancer-specific QoLQ were conducted in Asia (12 studies), followed by ten in Europe (32% out of total), two in North America (6%), two in Developed Asia and Pacific (6%), one in Latin America (3%) and one in Africa (3%). The EORTC series was the most frequently used questionnaire globally, with a high percentage of use in Asia and Europe. In contrast, North America used the FACT series of questionnaires in all the identified studies related with cancer. Figure 6 summarizes the use of cancer QoLQ and number of studies that appeared in each region.

## Discussion

Our review identified 14 general and 25 disease-specific QoLQ. The most-used QoLQ were the SF-36, EQ-5D and EORTC-QLQ. Commonly studied diseases were cancer and malnutrition. Only 33% of the studies focused on adults 65 years and older. Regional variation in QoLQ use was observed, with EQ-5D used more frequently in Europe and SF-36 more commonly used in North America.

Results of our systematic review found significant variation in use of QoLQ instruments for nutrition interventions. General questionnaires were widely utilized across various pathologies, age groups, and geographical locations, with the SF series and EQ-5D being the most prevalent. This finding is consistent with previous research by Haraldstad et al. [171]. Both instruments are short and contain simple, straightforward question items. Both are readily available in a variety of languages, and can be either self-administrated or by interview. This facilitates their use in nutrition interventions regardless of population characteristics. However, their simplicity may hinder capturing aspects of quality of life that are significant in specific population groups [172].

Among disease-specific questionnaires, a variety of instruments were also found. Cancer-specific QoLQ were widely used, and we identified several tools for assessing QoL in patients with cancer; EORTC-QLQ series, one of those Cancer-QoLQ, was the questionnaire most use among disease-specific QoLQ. In contrast, other pathologies were found to be assessed typically by general QoLQ, even though it has been reported that general QoLQ may be less sensitive to changes in disease or treatment compared to disease-specific instruments in several pathologies [173]. For example, the FACT-C questionnaire for patients with colorectal cancer includes questions about digestion, stomach cramping and the impact of an ostomy appliance [137]. This enables researchers to examine the ways an intervention may change how patients experience treatment and illness in greater depth than questionnaires focusing on general functionality and overall health. Additionally, disease-specific QoLQ may examine QoL domains not included in general QoLQ. For example, the EQ-5D, a general QoLQ, uses mobility, self-care, usual activities, pain/discomfort, and anxiety/depression domains whereas the IWQOL-Lite, a QoLQ assessing the impact of weight on adult QoL, assesses the domains of physical function, self-esteem, sexual life, public distress, and work. It is highly recommendable that researchers evaluate disease-specific QoLQ available to them and include such questionnaires as appropriate.

Given the additional information provided by disease-specific QoLQ, we were surprised that only eight studies used both general and disease-specific questionnaires. The use of these two types together has been recommended previously and yielded interesting results [174]. As a case in point, Ard et al. found that a nutrition intervention improved overall QoL as measured by the SF-36, as well as self-esteem as measured by the IWQOL [17]. Although additional information may be beneficial for a study, resources in any study are at a premium and the use of multiple QoLQ may challenge study budgets, while increasing the amount of time required for patients to participate in the study. Researchers should balance these costs against the potential benefits of better understanding how interventions impact patients’ lives and experiences.

This review also shows that QoLQs have been used to study the impact of nutrition interventions on quality of life of patients with a variety of illnesses. In particular, we found that studies of nutrition interventions and QoL focused heavily on cancer and malnutrition. This is not surprising since previous studies have reported the importance of evaluating QoL when determining nutritional status to tailor the nutritional intervention to the specific individual requirements of cancer [175] and malnourished patients [176]. Fewer studies examined nutrition interventions and QoL in patients with other illnesses such as diabetes or cardiovascular disease, even though the evidence shows a link between nutrition and QoL in patients with these pathologies [177, 178]. This suggests an opportunity for future research to expand the use of QoLQ in nutrition intervention studies, particularly in these diseases where nutrition interventions may have a significant impact on QoL.

Another focus of our study was nutrition interventions in specific age groups, especially in older adults, where nutrition interventions may be particularly impactful on their QoL [13, 179]. The aging process reduces appetite and individuals’ ability to ingest sufficient food to meet nutritional requirements, reducing physical and cognitive function, and QoL [9, 180–182]. Nutrition interventions assist older individuals in meeting nutritional requirements, maintaining physical and cognitive function, key components of QoL. However, we identified few studies focusing on this population, consistent with Arensberg et al. findings [183]. The absence or exclusion of older adults from clinical trials restricts data availability, forcing clinicians to make treatment decisions for older adults without adequate guidance [184]. For instance, only 4% of participants in cancer clinical trials conducted between 2005 and 2015 were aged over 80, whereas around 16% of individuals aged 80 or older in 2013 were diagnosed with cancer [185].

Promoting independence and healthy aging in a growing elderly population presents several key challenges. These include assessing the significance of factors such as nutrition in enhancing quality of life, developing effective interventions through research, and translating these findings into policies for implementation [183]. Future studies of nutrition interventions should focus on elderly populations and include QoL as an endpoint.

We also identify significant regional variation in QoLQ usage. EQ-5D questionnaires are more frequently used in Europe while the SF family of questionnaires are more commonly used in North America. The cause of this variation is beyond the scope of this project; however, it highlights the need for researchers and professional societies to develop harmonized guidelines for use of QoLQ in nutrition intervention studies. Such efforts would facilitate the comparison of nutrition interventions’ impact on QoL across studies.

## Conclusions

This review examined 116 articles that utilized QoLQ to ascertain how quality of life was measured in studies of nutritional intervention. We identified 39 different instruments used in studies from all parts of the world. Use of QoLQ to measure HRQoL is well established in the literature, with both general and disease-specific instruments being employed, but there is not a single dominant questionnaire in use. Instead, HRQoL instrument choice appears to be driven by the location in which the study takes place and the patient population being studied. Future researchers should consider these factors when selecting of a QoLQ for future nutritional intervention studies to facilitate comparability of results across studies. We also encourage researchers and professional societies to develop harmonized guidelines for use of QoL instruments for nutritional intervention studies with the aim to better understand the value and impact of nutrition interventions on quality of life of patients across different disease states and across different care settings.

### Supplementary Information

**Additional file 1: Supplemental Figure 1. **Boolean logic of database searches. **Supplemental Table 1.**  Compilation of included studies.

## Data Availability

Data will be made available upon reasonable request to the authors.

## Abbreviations

AQoL: Assessment of Quality of Life
EORTC: European Organisation for Research and Treatment
EQ-5D: EuroQol 5 dimensions; refers to a set of quality of life questionnaires. See Table 1
HRQoL: Health-related quality of life
IWQOL: Impact of Weight on QoL refers to a quality of life questionnaire. See Table 1
MeSH: Medical Subject Headings
PedsQL: Pediatric Quality of Life Inventory
PRISMA: Preferred Reporting Items for Systematic Reviews and Meta-Analyses
QoL: Quality of Life
QoLQ: Quality of Life Questionnaire
SF-36: Short-form, 36 questions; refers to a set of quality of life questionnaires. See Table 1.
WHO: World Health Organization
