# Supplementary material for: How quality of life is measured in studies of nutritional intervention: a systematic review
Source: Health Qual Life Outcomes. 2024 Jan 24;22:9. doi: 10.1186/s12955-024-02229-y (PMC10809546; doi:10.1186/s12955-024-02229-y)
Supplement: Supplementary file 1 — Additional file 1: Supplemental Figure 1. Boolean logic of database searches. Supplemental Table 1. Compilation of included studies. [file 12955_2024_2229_MOESM1_ESM.docx]

**Supplemental Figure 1.** Boolean logic of database searches

| AB, TI ("quality of life" OR "health-relate* quality of life" OR QoL OR HRQoL OR EuroQoL OR SF-36 OR QLQ-C30) AND ("diet$3 supplemention" OR "nutrition$2 counseling" OR "nutrition$2 intervention" OR "nutrition$2 therapy" OR "diet$3 intervention" OR "oral nutrition$2 supplement$5" OR ONS OR "home enteral" OR "home enteral nutrition" OR ONS OR "dietary patterns" OR "nutritional assessment" OR "oral supplement$5" OR "nutritional supplementation" OR "diet$3 intervention" OR "diet support") |
| --- |
| (Mesh("quality of life")) AND ((Mesh("Nutrition Therapy")) OR (Mesh("diet therapy"))) |

| **Supplemental Table 1.**  Compilation of included studies | | | | | | |
| --- | --- | --- | --- | --- | --- | --- |
| **Author (Year)** | **Country** | **Title** | **Journal** | **Age group** | **Pathology** | **QoLQ used** |
| Blondal et al., (2022) | Iceland | HOMEFOOD randomised trial e Six-month nutrition therapy improves quality of life, self-rated health, cognitive function, and depression in older adults after hospital discharge | Clinical Nutrition ESPEN | Older Adults | Malnourished. | EQ-5D-5L |
| Chen et al., (2022) | China | Effects of five-step nutritional interventions conducted by a multidisciplinary care team on gastroenteric cancer patients undergoing chemotherapy: a randomized clinical trial | Nutrition and Cancer | Adults | Cancer. | EORTC-QLQ-C30 |
| Folope et al., (2022) | France | Evaluation of a supervised adapted physical activity program associated or not with oral supplementation with arginine and leucine in subjects with obesity and metabolic syndrome: a randomized controlled trial | Nutrients | Adults | Metabolic Syndrome. | SF-36 |
| Gomez et al., (2022) | Colombia | A nutritionally focused program for community-living older adults resulted in improved health and well-being | Clinical Nutrition | Older Adults | Nonspecific. | EQ-5D-3L |
| Guo et al., (2022) | China | Non-protein energy supplement for malnutrition treatment in patients with chronic kidney disease | Asia Pacific Journal of Clinical Nutrition | Adults | Chronic kidney disease. Malnourished. | SF-36 |
| Hamidianshirazi et al., (2022) | Iran | Diet therapy along with Nutrition Education can Improve Renal Function in People with Stages 3-4 chronic kidney disease who do not have diabetes. (A randomized controlled trial) | The Nutrition Society Advancing Nutritional Science | Adults | Chronic kidney disease. | SF-12 |
| Hassanin et al., (2022) | Egypt | Implications of inappropriate prescription of oral nutritional supplements on the quality of life of cancer outpatients: a cross‑sectional comparative study | Supportive Care in Cancer | Adults | Cancer. | EORTC-QLQ-C30 |
| Huggins et al., (2022) | Australia | Effect of early and intensive telephone or electronic nutrition counselling delivered to people with upper gastrointestinal cancer on quality of life: a three-arm randomised controlled trial | Nutrients | Adults | Cancer. | EORTC-QLQ-C30 EQ-5D-5L |
| Kahleova et al., (2022) | USA | Nutrition for hospital workers during a crisis: effect of a plant-based dietary intervention on cardiometabolic outcomes and quality of life in healthcare employees during the COVID-19 pandemic | American Journal of Lifestyle Medicine | Adults | Nonspecific. | SF-36 |
| Lahiji et al., (2022) | Iran | Effectiveness of logotherapy and nutrition counseling on psychological status, quality of life, and dietary intake among breast cancer survivors with depressive disorder: a randomized clinical trial | Springer | Adults | Cancer. Mental issue | EORTC-QLQ-C30 EORTC-QLQ-BR23 |
| Long-Parma et al., (2022) | USA | Effect of an anti‑inflammatory dietary intervention on quality of life among breast cancer survivors | Springer | Adults | Cancer. | FACT-G FACT-B |

| **Table 1.** *(continued)* | | | | | | |
| --- | --- | --- | --- | --- | --- | --- |
| **Abbreviation** | **Country** | **Title** | **Journal** | **Age group** | **Pathology** | **QoLQ used** |
| Lu et al., (2022) | China | Analysis of the effect of exercise combined with diet intervention on postoperative quality of life of breast cancer patients | Computational and Mathematical Methods in Medicine | Adults | Cancer. Surgery. | EORTC-QLQ-C30 SF-36 |
| Mościcka et al., (2022) | Poland | Complex treatment of venous leg ulcers including the use of oral nutritional supplementation: results of 12-week prospective study | Advances in Dermatology and Allergology | Adults | Ulcer. | WHOQoL-BREF |
| Seemer et al., (2022) | Germany | Effects of an individualised nutritional intervention to tackle malnutrition in nursing homes: a pre‑post study | European Geriatric Medicine | Older Adults | Malnourished. | QUALIDEM |
| Sim et al., (2022) | South Korea | The effect of omega-3 enriched oral nutrition supplement on nutritional indices and quality of life in gastrointestinal cancer patients: a randomized clinical trial | Asian Pacific Journal of Cancer Prevention | Adults | Cancer. | EORTC-QLQ-C30 |
| Söderström et al., (2022) | Sweden | Oral nutritional supplement use is weakly associated with increased subjective health-related quality of life in malnourished older adults: a multicentre randomised controlled trial | British Journal of Nutrition | Older Adults | Malnourished. | EQ-5D-3L |
| Wu et al., (2022) | China | Role of nutritional support under clinical nursing path on the efficacy, quality of life, and nutritional status of elderly patients with Alzheimer’s disease | Evidence-Based Complementary and Alternative Medicine | Older Adults | Alzheimer. | WHOQoL-BREF |
| Yeung et al., (2022) | China | A nutritionally complete oral nutritional supplement powder improved nutritional outcomes in free-living adults at risk of malnutrition: a randomized controlled trial | International Journal of Environmental Research and Public Health | Adults | Malnourished. | |
| Zhu et al., (2022) | China | Effect of swallowing training combined with nutritional intervention on the nutritional status and quality of life of laryngeal cancer patients with dysphagia after operation and radiotherapy | Journal of oral rehabilitation | Adults | Cancer. Dysphagia | EORTC-QLQ-C30 |
| Ayça et al., (2021) | Turkey | Nutritional interventions improve quality of life of caregivers of children with neurodevelopmental disorders | Nutritional Neuroscience | Children | Mental issue. | WHOQoL-BREF |
| Barnes et al., (2021) | Australia | Efficacy of a school-based physical activity and nutrition intervention on child weight status: Findings from a cluster randomized controlled trial | Preventive Medicine | Children | Nonspecific. | PedsQL |
| Cramon et al., (2021) | Denmark | Individual nutritional intervention for prevention of readmission among geriatric patients—a randomized controlled pilot trial | BMC Public Health | Older Adults | Nonspecific. | EQ-5D-5L |
| de Sevilla et al., (2021) | Spain | Adherence to a lifestyle exercise and nutrition intervention in university employees during the COVID-19 pandemic: a randomized controlled trial | International Journal of Environmental Research and Public Health | Adults | Nonspecific. | SF-36 |
| Lin et al., (2021) | Taiwan | Effects of a food preparation program on dietary well-being for stroke patients with dysphagia | Medicine | Adults | Dysphagia. | WHOQoL-BREF SWAL-QOL |
| Meng et al., (2021) | China | Post-discharge oral nutritional supplements with dietary advice in patients at nutritional risk after surgery for gastric cancer: A randomized clinical trial | Clinical Nutrition | Adults | Cancer. Surgery. Malnourished. | EORTC-QLQ-C30 |
| Miao et al., (2021) | China | Effects of high-quality nursing in patients with lung cancer undergoing chemotherapy and related influence on self-care ability and pulmonary function | American Journal of Translational Research | Adults | Cancer. | EORTC-QLQ-C30 |
| Mousavi-Shirazi-Fard et al., (2021) | Iran | The effects of modified anti-inflammatory diet on fatigue, quality of life, and inflammatory biomarkers in relapsing-remitting multiple sclerosis patients: a randomized clinical trial | International Journal of Neuroscience | Adults | Multiple sclerosis. | MSQoL-54 |
| Munk et al., (2021) | Denmark | A multimodal nutritional intervention after discharge improves quality of life and physical function in older patients e a randomized controlled trial | Clinical Nutrition | Older Adults | Nonspecific. | EQ-5D-3L |
| Nguyen et al., (2021) | Vietnam | Nutrition intervention is beneficial to the quality of life of patients with gastrointestinal cancer undergoing chemotherapy in Vietnam | Cancer Medicine | Adults | Cancer. | EORTC-QLQ-C30 |
| Pascoe et al., (2021) | UK | Beta-hydroxy beta-methyl butyrate/arginine/ glutamine (HMB/Arg/Gln) supplementation to improve the management of cachexia in patients with advanced lung cancer: an open-label, multicenter, randomized, controlled phase II trial (NOURISH) | BMC Cancer | Adults | Cancer. | FAACT |
| Pinto et al., (2021) | Italy | QOLEC2: a randomized controlled trial on nutritional and respiratory counseling after esophagectomy for cancer | Supportive Care in Cancer | Adults | Cancer. Surgery. | EORTC-QLQ-C30 EORTC-QLQ-OES18 |
| Priscilla Silva de Souza et al., (2021) | Brazil | Nutritional intervention contributes to the improvement of symptoms related to quality of life in breast cancer patients undergoing neoadjuvant chemotherapy: a randomized clinical trial | Nutrients | Adults | Cancer. | EORTC-QLQ-C30 |
| Regueme et al., (2021) | France | Protein intake, weight loss, dietary intervention, and worsening of quality of life in older patients during chemotherapy for cancer | Supportive Care in Cancer | Older Adults | Cancer. Malnourished. | EORTC-QLQ-C30 |
| Reinders et al., (2021) | Netherlands and Finland | The cost effectiveness of personalized dietary advice to increase protein intake in older adults with lower habitual protein intake: a randomized controlled trial | European Journal of Nutrition | Older Adults | Nonspecific. | EQ-5D-5L |
| Wang et al., (2021) | Taiwan | Liquid combination of hyaluronan, glucosamine, and chondroitin as a dietary supplement for knee osteoarthritis patients with moderate knee pain | Medicine | Adults | Rheumatic disease. | SF-36 |
| Xie et al., (2021) | China | A randomized controlled trial of oral nutritional supplementation versus standard diet following McKeown minimally invasive esophagectomy in patients with esophageal malignancy: A pilot study | Annals of Translational Medicine | Adults | Cancer. Surgery. | EORTC-QLQ-C30 |
| Yixian et al. (2021) | Australia | Effect of Individual Nutrition Therapy and Exercise Regime on Gait Speed, Physical Function, Strength and Balance, Body Composition, Energy and Protein, in Injured, Vulnerable Elderly: A Multisite Randomized Controlled Trial (INTERACTIVE) | Nutrients | Older Adults | Nonspecific. | AQoL-4D |
| Zhang et al., 2021) | China | The effect of oral nutritional supplements on the nutritional status of community elderly people with malnutrition or risk of malnutrition | Asia Pacific Journal of Clinical Nutrition | Older Adults | Malnourished. | SF-36 |
| Ahmadi et al., (2020) | Iran | Fortified whey beverage for improving muscle mass in chronic obstructive pulmonary disease: A single-blind, randomized clinical trial | Respiratory Research | Adults | Pulmonary disease. | SGRQ |
| Chen et al., (2020) | USA | Home‑Based Physical Activity and Diet Intervention to Improve Physical Function in Advanced Liver Disease: A Randomized Pilot Trial | Springer | Adults | Liver disease. | SIP |
| Grönstedt et al., (2020) | Sweden | Effect of sit-to-stand exercises combined with protein-rich oral supplementation in older persons: the older person’s exercise and nutrition study | Journal of the American Medical Directors Association | Older Adults | Nonspecific. | EQ-5D-5L |
| Guerra-Sánchez et al., (2020) | Spain | Effect of a double nutritional intervention on the nutritional status, functional capacity, and quality of life of patients with chronic heart failure: 12-month results from a randomized clinical trial | Nutrición Hospitalaria | Adults | Heart failure. Malnourished. | MLHFQ |
| Ho et al., (2020) | Japan | Effects of dietary and physical activity interventions on generic and cancer-specific health-related quality of life, anxiety, and depression in colorectal cancer survivors: a randomized controlled trial | Springer | Adults | Cancer. | SF-12 FACT-C FACT-G |
| Huang et al., (2020) | China | A prospective randomized controlled trial on the value of prophylactic oral nutritional supplementation in locally advanced nasopharyngeal carcinoma patients receiving chemo-radiotherapy | Oral Oncology | Adults | Cancer. | EORTC-QLQ-C30 |
| Huarui et al., (2020) | China | Effects of overall enteral nutrition management overall on nutritional condition and life quality of NSCLC patients treated by apatinib combined with chemoradiotherapy | International Journal of Clinical and Experimental Medicine | Adults | Cancer. | SF-36 |
| Marchasso et al.,(2020) | Italy, Germany, France | Quality of Life: Psychological Symptoms-Effects of a 2-Month Healthy Diet and Nutraceutical Intervention; A Randomized, Open-Label Intervention Trial (RISTOMED) | Nutrients | Older Adults | Nonspecific. | SF-36 |
| Matthews et al., (2020) | Australia | Effects of a multicomponent exercise program combined with calcium-vitamin D3-enriched milk on health-related quality of life and depressive symptoms in older men: secondary analysis of a randomized controlled trial | European Journal of Nutrition | Older Adults | Nonspecific. | SF-36 |
| Miller et al., (2020) | USA | A randomized controlled trial testing the effectiveness of coping with cancer in the kitchen, a nutrition education program for cancer survivors | Nutrients | Adults | Cancer. | FACT-G7 |
| Mohd-Yusof et al., (2020) | Malaysia | Comparison of structured nutrition therapy for Ramadan with standard care in type 2 diabetes patients | Nutrients | Adults | Diabetes. | RV-DQOL |
| Nguyen et al., (2020) | Vietnam | Effectiveness of tailored dietary counseling in treating malnourished outpatients with chronic obstructive pulmonary disease: a randomized controlled trial | Journal of the academy of nutrition and dietetics | Adults | Pulmonary disease. Malnourished. | SGRQ |
| Poll et al., (2020) | Brazil | Impact of intervention on nutritional status, consumption of processed foods, and quality of life of adolescents with excess weight | Jornal de Pediatria | Children | Overweight. | PedsQL |
| Prasada et al., (2020) | USA | Implementation and impact of home-based cardiac rehabilitation in a veterans affair medical center | Military Medicine | Older Adults | Heart failure. | SF-36 |
| Rupnik et al., (2020) | Slovenia | Feasibility and safety of exercise training and nutritional support prior to hematopoietic stem cell transplantation in patients with hematologic malignancies | BMC Cancer | Adults | Cancer. | EORTC-QLQ-C30 |
| Sanz-Paris et al., (2020) | Spain | Impact of an oral nutritional protocol with oligomeric enteral nutrition on the quality of life of patients with oncology treatment-related diarrhea | Nutrients | Adults | Cancer. Malnourished. | NHP |
| Smith et al., (2020) | UK | Ready-made oral nutritional supplements improve nutritional outcomes and reduce health care use-a randomised trial in older malnourished people in primary care | Nutrients | Older Adults | Malnourished. | EQ-5D-5L |
| Strath et al., (2020) | USA | The effect of low-carbohydrate and low-fat diets on pain in individuals with knee osteoarthritis | Pain Medicine | Older Adults | Pain. Rheumatic disease. | KOOS QoL |
| van Beers et al., (2020) | Netherlands | Clinical outcome and cost-effectiveness of a 1-year nutritional intervention programme in COPD patients with low muscle mass: The randomized controlled NUTRAIN trial | Clinical Nutrition | Adults | Pulmonary disease. | EQ-5D-3L |
| van der Werf et al., (2020) | Netherlands | The effect of nutritional counseling on muscle mass and treatment outcome in patients with metastatic colorectal cancer undergoing chemotherapy: A randomized controlled trial | Clinical Nutrition | Adults | Cancer. | EORTC-QLQ-C30 |
| Bo et al., (2019) | China | A high whey protein, vitamin D and E supplement preserves muscle mass, strength, and quality of life in sarcopenic older adults: A double-blind randomized controlled trial | Clinical Nutrition | Older Adults | Sarcopenia. | SF-36 |
| Guerra-Sanchez et al., (2019) | Spain | Nutritional intervention in patients with heart failure. 3 month results of a clinical trial | Nutrición Clínica y Dietética Hospitalaria | Adults | Heart failure. Malnourished. | MLHFQ |
| Hagberg et al., (2019) | Sweden | Cost-effectiveness and quality of life of a diet intervention postpartum: 2-year results from a randomized controlled trial 11 medical and health Sciences 1117 public health and health services | BMC Public Health | Adults | Nonspecific. | SF-36 EQ-5D-3L |
| Katherine et al., (2019) | Australia | The effect of a pilot dietary intervention on pain outcomes in patients attending a tertiary pain service | Nutrients | Adults | Pain. | SF-36 |
| Khammassi et al., (2019) | France | Health-related quality of life and perceived health status of adolescents with obesity are improved by a 10-month multidisciplinary intervention | Physiology & Behavior | Children | Overweight. | SF-36 |
| Kobayashi et al., (2019) | Japan | Bifidobacterium Breve A1 supplementation improved cognitive decline in older adults with mild cognitive impairment: an open-label, single-arm study | The Journal of Prevention of Alzheimer’s Disease | Older Adults | Mental issue. | POMS2 |
| Kommers et al., (2019) | Brazil | Effects of probiotic use on quality of life and physical activity in constipated female university students: a randomized, double-blind placebo-controlled study | The journal of alternative and complementary medicine | Adults | Intestinal constipation. | PAC-QoL |
| Matia-Martin et al., (2019) | Spain | Effectiveness of an oral diabetes-specific supplement on nutritional status, metabolic control, quality or life, and functional status in elderly patients. A multicentre study | Clinical Nutrition | Older Adults | Diabetes. | EQ-5D-3L |
| Najafi et al., (2019) | Iran | Randomized study of the effect of dietary counseling during adjuvant chemotherapy on chemotherapy induced nausea and vomiting, and quality of life in patients with breast cancer | Nutrition and Cancer | Adults | Cancer. | EORTC-QLQ-C30 |
| Parletta et al., (2019) | Australia | A Mediterranean-style dietary intervention supplemented with fish oil improves diet quality and mental health in people with depression: A randomized controlled trial(HELFIMED) | Nutritional Neuroscience | Adults | Mental issue. | aQoL-8D |
| Pezhman et al., (2020) | Iran | The effect of nutritional education and continuous monitoring on clinical symptoms, knowledge, and quality of life in patients with cirrhosis | Gastroenterology and Hepatology From Bed to Bench. | Adults | Liver disease. | CLDQ |
| Seong-Hyeon et al., (2019) | Korea | The effect of nutrition intervention with oral nutritional supplements on pancreatic and Bile duct cancer patients undergoing chemotherapy | Nutrients | Adults | Cancer. | EORTC-QLQ-C30 |
| Wills et al., (2019) | USA | Nutritional counseling with or without mobile health technology: a randomized open-label standard-of-care-controlled trial in ALS | BMC Neurology | Adults | Amyotrophic Lateral Sclerosis. | PROMIS SF |
| Zhu et al., (2019) | China | Effect of oral nutritional supplementation on the post-discharge nutritional status and quality of life of gastrointestinal cancer patients after surgery: a multi-center study | Asia Pacific journal of clinical nutrition | Adults | Cancer. Surgery. | EQ-5D-3L |
| Cereda et al., (2018) | Italy | Nutritional counseling with or without systematic use of oral nutritional supplements in head and neck cancer patients undergoing radiotherapy | Radiotherapy and Oncology | Adults | Cancer. | EORTC-QLQ-C30 |
| de Luis et al., (2018) | Spain | AdNut study: effectiveness of a high calorie and protein oral nutritional supplement with β‑hydroxy‑β‑methylbutyrate in an older malnourished population in usual clinical practice | European Geriatric Medicine | Older Adults | Malnourished. | EQ-5D-3L |
| Esperanza et al., (2018) | Spain | Pulmonary rehabilitation only versus with nutritional supplementation in patients with bronchiectasis | Pulmonary Rehabilitation | Adults | Bronchiectasis. | QoL-B |
| Froghi et al.,(2018) | UK | A randomised trial of post-discharge enteral feeding following surgical resection of an upper gastrointestinal malignancy | Clinical Nutrition | Adults | Cancer. Surgery. | EQ-5D-5L EORC-QLQ-OES18 |
| Gomi et al., (2018) | Japan | Bifidobacterium bifidum YIT 10347 fermented milk exerts beneficial effects on gastrointestinal discomfort and symptoms in healthy adults: A double-blind, randomized, placebo-controlled study | American Dairy Science Association | Adults | Nonspecific. | SF-36 |
| Jiang et al., (2018) | China | Benefits of oral nutritional supplements in patients with locally advanced nasopharyngeal cancer during concurrent chemoradiotherapy: an exploratory prospective randomized trial | Nutrition and Cancer | Adults | Cancer. | EORTC-QLQ-C30 EORTC QLQ-H&N35 |
| Kong et al., (2018) | Korea | Effect of perioperative oral nutritional supplementation in malnourished patients who undergo gastrectomy: A prospective randomized trial | Surgery | Adults | Cancer. Malnourished. Surgery. | EORTC-QLQ-C30 |
| Langabeer et al., (2018) | USA | Effects of a community population health initiative on blood pressure control in latinos | American Heart Association | Adults | Hypertension. | EQ-5D VAS |
| Lindqvist et al., (2018) | Sweden | Influence of Blue Mussel (Mytilus edulis) Intake on Disease Activity in Female Patients with Rheumatoid Arthritis: The MIRA Randomized Cross-Over Dietary Intervention | Nutrients | Adults | Rheumatic disease. | SF-36 |
| Magalhães et al., (2018) | Brazil | The impact of a nutrition intervention program targeting elderly people with chronic kidney disease | Ciencia e Saude Coletiva | Older Adults | Chronic kidney disease. | WHOQoL-BREF |
| Salamon et al., (2018) | Australia | Oral nutritional supplementation in patients undergoing peritoneal dialysis: a randomised, crossover pilot study | Journal of Renal Care | Adults | Chronic kidney disease. Malnourished. | KDQOL-SF EQ-5D-5L |
| Towery et al., (2018) | USA | Chronic musculoskeletal pain and function improve with a plant-based diet | Complementary Therapies in Medicine | Adults | Pain. | SF-36 |
| Ulian et al., (2018) | Brazil | Effects of a new intervention based on the Health at Every Size approach for the management of obesity: The “Health and Wellness in Obesity” study | PLOS ONE | Adults | Overweight. | WHOQoL-BREF |
| Von-Berens et al., (2018) | Sweden | Effect of exercise and nutritional supplementation on health-related quality of life and mood in older adults: The VIVE2 randomized controlled trial | BMC Geriatrics | Older Adults | Nonspecific. | SF-36 |
| Whybird et al., (2018) | Australia | Promoting quality of life and recovery in adults with mental health issues using exercise and nutrition intervention | International Journal of Mental Health | Adults | Mental issue. | AQoL-8D |
| Wyers et al., (2018) | The Netherlands | Efficacy of nutritional intervention in elderly after hip fracture: a multicenter randomized controlled trial | Journals of Gerontology: Medical Sciences | Older Adults | Bone fracture. | EQ-5D-3L |
| Young et al., (2018) | Australia | Improving nutritional discharge planning and follow up in older medical inpatients: Hospital to Home Outreach for Malnourished Elders | Nutrition & Dietetics | Older Adults | Malnourished. | AQoL-6D |
| Andersson et al., (2017) | Norway | Effect on body weight, quality of life and appetite following individualized, nutritional counselling to home-living Elderly after rehabilitation - an open randomized trial | The journal of nutrition, health & aging | Older Adults | Malnourished. | EQ-5D-3L |
| Ard et al., (2017) | USA | Effects of calorie restriction in obese older adults: the CROSSROADS randomized controlled trial | Journals of Gerontology: Medical Sciences | Older Adults | Overweight. | SF-36 IWQOL-Lite |
| Micka et al., (2017) | Germany | Effect of consumption of chicory inulin on bowel function in healthy subjects with constipation: a randomized, double-blind, placebo-controlled trial | International Journal of Food Sciences and Nutrition | Adults | Intestinal constipation. | PAC-QoL |
| Parsons et al., (2017) | UK | Oral nutritional supplements in a randomised trial are more effective than dietary advice at improving quality of life in malnourished care home residents | Clinical Nutrition | Older Adults | Malnourished. | EQ-5D-3L |
| Saboya et al., (2017) | Brazil | Lifestyle Intervention on Metabolic Syndrome and its Impact on Quality of Life: A Randomized Controlled Trial | Sociedade brasileira de cardiologia | Adults | Metabolic Syndrome. | SF-36 |
| Sammarco et al., (2017) | Italy | Evaluation of Hypocaloric Diet with Protein Supplementation in Middle-Aged Sarcopenic Obese Women: A Pilot Study | The European Journal of Obesity | Adults | Sarcopenia. Overweight. | SF-36 |
| Sharma et al. (2017) | Australia | Investigation of the benefits of early malnutrition screening with telehealth follow up in elderly acute medical admissions | Oxford | Older Adults | Malnourished. | EQ-5D-5L |
| Werner et al., (2017) | Germany | Dietary supplementation with n-3-fatty acids in patients with pancreatic cancer and cachexia: marine phospholipids versus fish oil - a randomized controlled double-blind trial | Lipids in Health and Disease | Adults | Cancer. | EORTC-QLQ-C30 EORTC-QLQ-PAN26 |
| Zeng et al., (2017) | China | Home enteral nutrition's effects on nutritional status and quality of life after esophagectomy | Asia Pacific journal of clinical nutrition | Adults | Cancer. Surgery. | EORTC-QLQ-C30 EORTC-QLQ-OES18 |
| Beck et al. ,(2016) | Denmark | Multidisciplinary nutritional support for undernutrition in nursing home and home-care: A cluster randomized controlled trial | Nutrition Journal | Older Adults | Malnourished. | EQ-5D-3L |
| Geaney et al. (2016) | Ireland | The effect of complex workplace dietary interventions on employees' dietary intakes, nutrition knowledge and health status: a cluster controlled trial | Preventive Medicine | Adults | Nonspecific. | EQ-5D-5L |
| Holst et al., (2016) | Denmark | Functional training and timed nutrition intervention in infectious medical patients | European Journal of Clinical Nutrition | Adults | Infectious. | EQ-5D-3L |
| Ma et al., (2016) | USA | Pilot randomised trial of a healthy eating behavioural intervention in uncontrolled asthma | European Respiratory Journal | Adults | Asthma. | MiniAQLQ |
| Sari et al., (2016) | Turkey | The effect of glutamine and arginine-enriched nutritional support on quality of life in head and neck cancer patients treated with IMRT | Clinical Nutrition ESPEN | Adults | Cancer. | EORTC-QLQ-C30,  EORTC-QLQ-H&N35  EORTC-QLQ-OES18 |
| Visvanathan et al., (2016) | Australia | The randomized control trial of the effects of testosterone and a nutritional supplement on hospital admissions in undernourished, community dwelling, older people | The Journal of Nutrition, Health and Aging | Older Adults | Malnourished. | SF-36 |
| Abizanda et al., (2015) | Spain | Effects of an oral nutritional supplementation plus physical exercise intervention on the physical function, nutritional status, and quality of life in frail institutionalized older adults: The ACTIVNES study | Journal of the American Medical Directors Association | Older Adults | Nonspecific. | EQ-5D-3L |
| Agarwal et al., (2015) | USA | A multicenter randomized controlled trial of a nutrition intervention program in a multiethnic adult population in the corporate setting reduces depression and anxiety and improves quality of life: The GEICO study | The science of Lifestyle Change | Adults | Diabetes. | SF-36 |
| Assaf et al., (2015) | USA | Low-fat dietary pattern intervention and health-related quality of life: the women’s health initiative randomized controlled dietary modification trial | Academy of Nutrition and Dietetics | Older Adults | Nonspecific. | SF-36 |
| Kwon et al., (2015) | Japan | Effects of a combined physical training and nutrition intervention on physical performance and health-related quality of life in prefrail older women living in the community: a randomized controlled trial | Journal of the American Medical Directors Association | Older Adults | Nonspecific. | SF-36 |
| Vivanti et al., (2015) | Australia | Emergency department malnutrition screening and support model improves outcomes in a pilot randomised controlled trial | British medical journal | Older Adults | Malnourished. | EQ-5D-5L |
| Costan et al., (2014) | Romania | Vitamin D fortified bread improves pain and physical function domains of quality of life in nursing home residents | Journal of Medicinal Food | Older Adults | Nonspecific. | EQ-5D-3L QUALEFFO-41 |
| Poulsen et al., (2014) | Denmark | Randomized trial of the effects of individual nutritional counseling in cancer patients | Clinical Nutrition | Adults | Cancer. | EORTC-QLQ-C30 |
| Sheard et al., (2014) | Australia | Improved nutritional status is related to improved quality of life in Parkinson’s disease | BMC Neurology | Adults | Parkinson. | PDQ-39 |
| Li et al., (2013) | Canada | Impact of a trimodal rehabilitation program on functional recovery after colorectal cancer surgery: A pilot study | Surgical Endoscopy | Adults | Cancer. Surgery. | SF-36 |
| Stange et al., (2013) | Germany | Effects of a low-volume, nutrient- and energy-dense oral nutritional supplement on nutritional and functional status: a randomized, controlled trial in nursing home residents | The Journal of Post-Acute and Long -Term Care Medicine | Older Adults | Nonspecific. | QUALIDEM |
| Casas et al., (2012) | Spain | Adapted ice cream as a nutritional supplement in cancer patients: impact on quality of life and nutritional status | Clinical and Translational Oncology | Adults | Cancer. Malnourished. | EORTC-QLQ-C30 |
| Dal Negro et al., (2012) | Italy | Essential amino acid supplementation in patients with severe COPD: A step towards home rehabilitation | Monaldi Archives for Chest Disease | Older Adults | Pulmonary disease. | SGRQ |
| **Abbreviations: AQoL-4D:** Assessment of Quality of Life-4 Dimension; **AQoL-6D:** Assessment of Quality of Life-6 Dimension; **AQoL-8D:** Assessment of Quality of Life-8 Dimension; **CLDQ:** Chronic Liver Disease Questionnaire; **EORTC:** European Organization for Research and Treatment of Cancer; **EORTC-QLQ-BR23:** EORTC-Breast Cancer Module; **EORTC-QLQ-C30.** EORTC- General Cancer Questionnaire; **EORTC-QLQ-H&N35:** EORTC- Head & Neck Cancer Module; **EORTC-QLQ-OES18:** EORTC- Oseophageal Cancer Module; **EQ-5D-VAS:** EuroQol– 5 Dimension Visual Analog Scale; **EQ-5D-3L:** 3-level EuroQol– 5 Dimension; **EQ-5D-5L:** 5-level EuroQol– 5 Dimension; **FAACT:** Functional Assessment of Anorexia/Cachexia Treatment; **FACT-B:** The Functional Assessment of Cancer Therapy – Breast; **FACT-C:** Functional Assessment of Cancer Therapy – Colorectal; **FACT-G:** Functional Assessment of Cancer Therapy – General; **FACT-G7:** Functional Assessment of Cancer Therapy – General – 7 Item Version; **IWQOL-Lite: Impact of** Weight on Quality of Life-Lite; **KDQOL-SF:** Kidney Disease Quality of Life Short Form; **KOOS:** Knee Injury and Osteoarthritis Outcome Score; **MiniAQLQ:** Mini Asthma Quality of Life Questionnaire; **MLHFQ:** Minnesota Living With Heart Failure Questionnaire; **MSQoL-54:** Multiple Sclerosis Quality of Life-54; **NHP:** Nottingham Health Profile; **PAC-QoL:** Patient Assessment Of Constipation Quality of Life; **PAN26:** EORTC Pancreatic Cancer Module; **PDQ-39:** The Parkinson's Disease Questionnaire; **PedsQoL:** Pediatric Quality of Life Inventory; **POMS2:** Profile of Mood States Second Edition; **PROMIS SF:** Patient Reported Outcomes Measurement Information System – Short Form;  **QoL-B:** Quality of Life Questionnaire-Bronchiectasis; **QUALEFFO-41:** Quality of Life Questionnaire of the European Foundation for Osteoporosis; **QUALIDEM:** Quality of Life for People with Dementia; **RV-DQOL:** Revised Version of Diabetes Quality of Life Instrument; **SF-12:** Short Form -12; **SF-36:** Short Form -36; **SGRQ:** St. George's Respiratory Questionnaire; **SIP:** Sickness Impact Profile; **SWAL-QOL:** Swallowing Quality of Life questionnaire; **WHOQoL-BREF:** World Health Organization Quality of Life Scale-BREF | | | | | | |
